# PLAS-20k: Extended Dataset of Protein-Ligand Affinities from MD Simulations for Machine Learning Applications

**DOI:** 10.1038/s41597-023-02872-y

**Published:** 2024-02-09

**Authors:** Divya B. Korlepara, Vasavi C. S., Rakesh Srivastava, Pradeep Kumar Pal, Saalim H. Raza, Vishal Kumar, Shivam Pandit, Aathira G. Nair, Sanjana Pandey, Shubham Sharma, Shruti Jeurkar, Kavita Thakran, Reena Jaglan, Shivangi Verma, Indhu Ramachandran, Prathit Chatterjee, Divya Nayar, U. Deva Priyakumar

**Affiliations:** 1 IHub-Data, International Institute of Information Technology, Hyderabad, 500032 India; 2 Divison of Physics, School of Advanced Sciences, Vellore Institute of Technology, Chennai, 600127 India; 3 Department of Artificial Intelligence, School of Artificial Intelligence, Amrita Vishwa Vidyapeetham, Bengaluru, 560035 India; 4 Centre for Computational Natural Sciences and Bioinformatics, International Institute of Information Technology, Hyderabad, 500032 India; 5 Department of Materials Science and Engineering, Indian Institute of Technology Delhi, Hauz Khas, New Delhi 110016 India

**Keywords:** Data publication and archiving, Virtual screening

## Abstract

Computing binding affinities is of great importance in drug discovery pipeline and its prediction using advanced machine learning methods still remains a major challenge as the existing datasets and models do not consider the dynamic features of protein-ligand interactions. To this end, we have developed PLAS-20k dataset, an extension of previously developed PLAS-5k, with 97,500 independent simulations on a total of 19,500 different protein-ligand complexes. Our results show good correlation with the available experimental values, performing better than docking scores. This holds true even for a subset of ligands that follows Lipinski’s rule, and for diverse clusters of complex structures, thereby highlighting the importance of PLAS-20k dataset in developing new ML models. Along with this, our dataset is also beneficial in classifying strong and weak binders compared to docking. Further, OnionNet model has been retrained on PLAS-20k dataset and is provided as a baseline for the prediction of binding affinities. We believe that large-scale MD-based datasets along with trajectories will form new synergy, paving the way for accelerating drug discovery.

## Background & Summary

High-throughput screening plays a crucial role in the drug discovery process. However, this approach to identifying lead molecules is time-consuming and labour-intensive. On the other hand, computational methods offer a promising solution by significantly reducing the cost, time, and resources required for physical experiments in screening potential hit molecules. High-throughput docking and molecular dynamics (MD) simulations provide an appealing virtual screening approach to expedite the discovery of biologically active hit compounds^1^. Despite the advantages of these methods, certain limitations and drawbacks still exist in docking. These include a restricted sampling of both protein and ligand conformation during pose prediction and the use of approximated scoring functions that often yield docking scores with poor correlation to experimental binding affinities^2^. On the other hand, MD simulations offer several benefits in investigating the structural and dynamical properties of a Protein-Ligand (PL) system and accurately predicting binding affinities. However, screening of umpteen molecules consumes prohibitively expensive computational resources rendering the prediction of binding affinity (MD based) on a large scale infeasible^3^.

In recent years, machine learning (ML) has emerged as a powerful tool to accelerate various aspects of drug development^4^. ML has already shown to be successful in the hunt for antibiotics^5^, drug re-purposing for emerging diseases^6,7^, virtual screening^8,9^, bio-molecular interactions, prediction of binding site and protein folding^10–14^. Notably, enormous ML models have been developed to predict PL binding affinity^15^. These data-driven approaches have been successful in attaining a high level of accuracy by learning the binding modes directly from rapidly growing experimental three-dimensional (3D) PL structural data deposited in Protein Data Bank (PDB)^16,17^. Numerous attempts have been made to enhance the performance of machine learning (ML) models through different types of encoding, topology, spectral sequence, and atom pairs. These approaches have predominantly relied on feature engineering from static 3D structures^18^. However, this static picture of PL interactions often lacks dynamic features. Incorporating dynamic properties can provide crucial insights into bio-molecular processes such as protein folding, conformational changes, and ligand binding. In addition, considering dynamic features can help address fundamental questions related to binding affinity and specificity^19,20^. The greatest strength of MD simulations lies in their ability to reveal dynamic effects of the bio-molecules that go beyond the experimentally determined structures available in PDB^21,22^. Furthermore, MD simulations capture the interactions and energy exchanges between the protein, ligand (solute), and solvent(water, buffer ions) to dictate the binding event through both long-range and short-range interactions^23–26^. While existing ML models have shown promise in predicting binding affinity, they often rely on training datasets composed of only a few hundred static binding poses of PL complexes. With the continuous growth in the number of ligands and proteins, there is an increasing demand for massive and dynamic data to improve the ML model′s accuracy in predicting binding affinities.

By integrating MD simulations with ML techniques, researchers can leverage the dynamic nature of biomolecular systems and incorporate a broader range of data, leading to more accurate and reliable predictions of binding affinities. The combination of MD simulations and ML holds great potential for accelerating drug discovery efforts in an ever-expanding chemical space. To this end, in our previous work, we developed an MD-based dataset called PLAS-5k^27^. This dataset included binding affinities averaged over conformations of each of 5000 PL complexes, representing various classes of enzymes. In addition to the binding affinities, the dataset also included energy components contributing to the binding free energy.

When attempting to accurate prediction of PL interactions through ML models, a labyrinth of interactions needs to be accounted for. In continuation to our previous dataset, the current work focuses on expanding heterogeneous proteins and a large spectrum of ligand types, including small organic molecules and peptides. The extended dataset, encompasses 19,500 PL structures, providing protein-ligand affinities and non-covalent interaction components, along with accompanying trajectories suitable for machine learning applications.

The creation of the PLAS dataset was primarily motivated by the need for high-quality datasets that can support the development of advanced algorithms and drive significant advancements in drug development. The PLAS-20k dataset comprises a diverse collection of protein-ligand (PL) complexes, providing a valuable resource for researchers in the field. To assess the performance of calculated binding affinities, we conducted comparisons by calculating correlation coefficients between experimentally determined values and the affinities obtained through molecular mechanics/Poisson-Boltzmann surface area (MMPBSA) and docking methods. This evaluation allowed us to validate the accuracy and reliability of the computational approaches employed. Based on the experimental binding affinities within the PLAS-20k dataset, we categorized the complexes into strong binders (SB) and weak binders (WB). This classification helps to differentiate between PL complexes with high and low affinities, providing valuable insights into the range of binding strengths within the dataset. Furthermore, we assessed the ligand’s adherence to Lipinski’s Rule of 5, which offers insights into their drug-like properties. As a baseline for comparison, we retrained the OnionNet framework using our dataset. The availability of large datasets is often considered essential for successful deep learning applications. Thus, we believe that the PLAS-20k dataset will serve as a catalyst for the development of data-driven methods in various drug design tasks, including hit identification, lead optimization, and de novo molecular design. By providing a comprehensive and diverse dataset, the PLAS-20k dataset empowers researchers to more effectively explore and apply data-driven approaches, leading to advancements in drug discovery and design processes. The dataset′s availability will drive further innovation and contribute to significant progress in the field of drug development.

## Methods

### Data Curation

In this article, we have chosen a set of 14,500 complexes from the Protein Data Bank (PDB)^17^, expanding upon our previous PLAS-5k^27^ dataset. The selection criteria for these complexes focused on proteins that are complex with small molecules (ligands) or peptides.

### Dataset Preparation

**Fig. 1:**
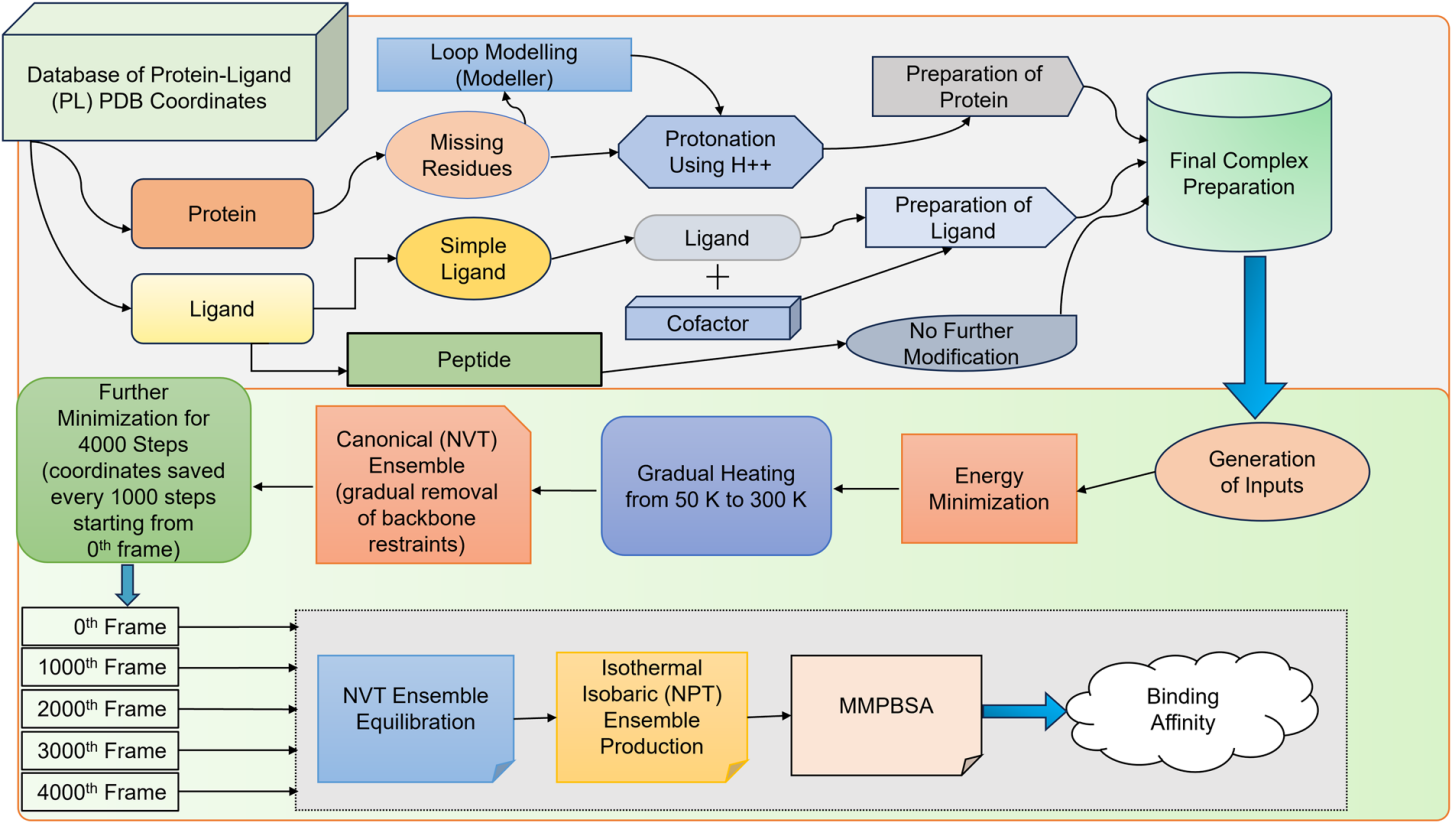
Flowchart corresponding to the system-setup and simulation protocol^27^.

We followed the preprocessing and calculation protocol similar to previous work^27^, in our current study. A brief account of the methods is given here. The initial structures of the complexes were taken from PDB^17^. Protein chains with missing residues were modelled as loop regions using UCSF Chimera^28,29^. Further, the protein chains were protonated at a physiological pH, 7.4 using H++ server^30^. The tleap program of ambertools^31,32^ was used to build the input files of each complex system (protein-ligand, cofactors and crystal water molecules) files required for MD simulations. The crystal waters were modelled using a TIP3P force field^33^ The proteins were modelled using Amber ff14SB force field^34^ in the all-atom model, and parameters of the ligand and cofactors were taken from General AMBER force field (GAFF2)^35^ using antechamber program^36^. Each complex was solvated in an orthorhombic TIP3P water box with a 10 Å extension from the protein surface. More detailed information on the dataset preparation is discussed in our earlier work with 5k complexes^27^ and the flowchart for data preparation is shown in Fig. 1. The counter ions were added to maintain the charge neutrality of the system.

MD simulations were performed using OpenMM 7.2.0 program^37^. The simulation protocol involved several steps as described below. To initiate the simulations, we performed a minimization process using the L-BFGS minimizer with a harmonic potential applied to the atoms of the protein backbone. The force constant for this potential was set to 10 kcal/mol/Å^2^. The minimization consisted of 1000 steps, and after every 10 steps, the restraint force on the backbone atoms was reduced by half. Subsequently, an additional 1000 steps of minimization were conducted after removing the harmonic potential entirely.

During the simulation, a time step of 2 fs was used, and constraints were applied to the bonds involving hydrogen atoms. We implemented a Langevin thermostat with a friction coefficient of 5 ps^−1^ to maintain the temperature. The system was gradually heated from an initial temperature of 50 K to the target temperature of 300 K, increasing by 1 K every 100 steps (200 fs). The backbone atoms of the protein were restrained using harmonic potentials during this heating process. Once the target temperature was reached, the simulations were performed for 1 ns in the NVT ensemble.

After equilibration, the final coordinates have been subjected to a further 4000 steps minimization. The coordinates were saved every 1000 steps starting from zero-th frame. Thereby five independent minimized conformations have been obtained to start the production runs. In the following step, each of these minimized coordinates were equilibrated in NVT ensemble at 300 K and 1 atm for 2 ns. Finally, a production run of 4 ns in NPT ensemble is performed using a Langevin thermostat and Monte Carlo barostat. Each of these trajectories (corresponding to each PLC) are saved every 100 ps for post-processing analysis (corresponding simulation protocol schematics provided in Fig. 1).

MD trajectories from five independent simulations were used to calculate the binding affinity using MMPBSA (Molecular-Mechanics Poisson Boltzmann Surface Area) method. In computing the binding affinity with MMPBSA, we used a single trajectory approach (receptor and ligand contributions were computed from each individual trajectories (and separately obtained from all five trajectories) for each PLC respectively. We considered two explicit water molecules near the active site. The binding affinity is calculated as follows:

1 $$\Delta {G}_{MMPBSA}=\Delta {E}_{MM}+\Delta {G}_{Sol}$$

Electrostatic interaction energy Δ*E*_*ele*_, and Van der Waals interaction energy Δ*E*_*vdw*_ contributes to Δ*E*_*MM*_ (Eq. (2)) and Δ*G*_*Sol*_, is defined as sum of polar Δ*G*_*pol*_, and non-polar contributions Δ*G*_*np*_ (Eq. (3))

2 $$\Delta {E}_{MM}=\Delta {E}_{ele}+\Delta {E}_{vdw}$$

3 $$\Delta {G}_{Sol}=\Delta {G}_{pol}+\Delta {G}_{np}$$

### Docking Methodology

Like our previous work^27^, we conducted docking studies using AutoDock Vina^38^ for structures with experimentally known binding affinities. Crystal structures for all protein-ligand (PL) complexes were sourced from the PDB database and refined by eliminating heteroatoms. Hydrogen atoms were subsequently added, and Kollman charges were assigned to the protein structures. For ligands, Gasteiger partial atomic charges were assigned, and all flexible torsion angles were defined using AUTOTORS. We discretized the active site of each target through a grid box (centered over the active site) and carried out docking calculations using the default parameters.

## Data Records

All data for all complexes can be accessed through figshare^39^.

## Technical Validation

### Usage Notes

In addition to the dataset version, PLAS-20k is also available publicly at (https://healthcare.iiit.ac.in/d4/plas20k/plas20k.html). The list of PDB ids that are part of PLAS-20k is provided and can be downloaded from the website. The PDB id search icon in the database opens a specific 3D structure along with energy components (Van der Waals interaction energy, electrostatic energy, polar and non-polar solvation free energies in conjunction with binding affinity) from the MD trajectories using the MMPBSA method. An example of HIV-1 protease complex (PDB id: 1hxw) is shown in Supplementary Figure S1.

### Molecular Heterogeneity of PLAS-20k

To characterize the extent of diversity of PLAS-20k over PLAS-5k (in terms of eminent molecular properties), we have undertaken a t-SNE (t-distributed stochastic neighbor embedding) distribution analyses over the PLAS-5k, and PLAS-20k datasets (Figure S2). The non-linear molecular properties were fetched from corresponding SMILES strings of the ligands, evidently including the Lipinski’s rule of 5. Interestingly, we find that the t-SNE distribution cover more sample space for PLAS-20k over PLAS-5k. This underscores the fact that the current results are based on a dataset with additional diversity of PLAS-20k over its predecessor (PLAS-5k).

### Overall Structures of the Protein-Ligand Complexes

Though there are a lot of advances in predicting PL binding affinity through machine learning methods, the incorporation of receptor flexibility remains a major bottleneck. In the present work, we propose a novel dataset based on binding affinities of PL complexes retrieved from MD simulations. The binding affinities were calculated by considering the flexibility of both protein and ligand. The simulated complexes were validated by calculating the RMSD with respect to the experimental structure. The protein structures were superimposed to calculate RMSD of protein and ligand. These calculations have been performed over 200 frames (40 from each simulation trajectory) and the corresponding distributions are shown in Supplementary Figure S3. The long tails of RMSD distributions of protein and ligand are evident due to the flexibility of the complex during the simulations.

### Comparison of experimental vs computed binding affinities

Experimentally, the binding affinity of a protein-ligand complex is expressed in terms of dissociation constant (K_*d*_) or inhibition constant (K_*i*_). This experimentally determined binding equilibrium constant is related to binding free energy as,

4 $$\Delta {G}_{expt}=-{k}_{B}T\,{ln}\,{K}_{i}=-{k}_{B}T\,{ln}\,(1/{K}_{d})$$

**Fig. 2:**
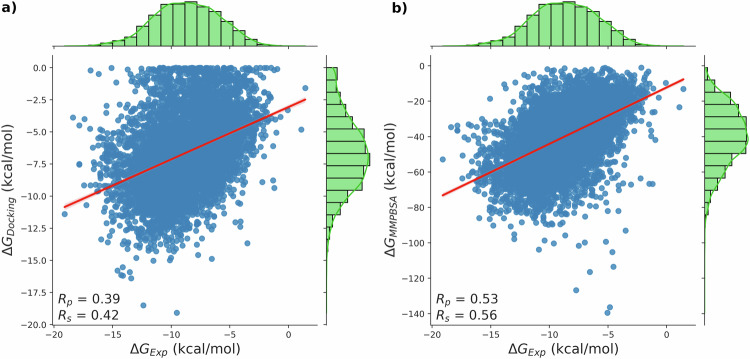
Correlation plots between the experimental and calculated binding affinities for a subset with 6622 (includes 2000 data points from PLAS-5k dataset^27^) pdbids. The calculated binding affinities are calculated (**a**) using Auto-dock Vina, and (**b**) using MMPBSA.

In this work, for a comparison study, we selected a subset of 6622 complexes of the PLAS-20k dataset, whose experimental binding affinities are available. To assess the performance of our dataset, the Pearson correlation coefficient (R_*p*_) and Spearman rank correlation coefficient (R_*s*_) were calculated. Both these correlation coefficients showed that, studies based on MMPBSA have superior performance with (R_*p*_) of 0.53 and (R_*s*_) of 0.56 compared to docking studies whose (R_*p*_) & (R_*s*_) are 0.39 and 0.41 respectively. The corresponding plots are shown in Fig. 2. The results highlight the importance of considering both protein and ligand flexibility. We expect that ML-based scoring functions developed using the PLAS-20k dataset could be more reliable than classical scoring functions. The distribution of the calculated binding affinity is shown in Supplementary Figure S4.

### Classification of Binders

Drug discovery is the process by which lead molecules are identified by screening chemical space based on binding affinity. The existing ML models or scoring functions were formulated based on several assumptions but they still have certain limitations. Mostly, researchers are interested in identifying only strong binders (SB), and one of the major reasons for neglecting weak binding molecules in drug discovery is because of its cross reactivity^40,41^. However, these weak binders (WB) are also equally important as they play a key role in fragment-based drug design^42^ and they serve as a foundation towards the development of more potent and selective drug candidates with improved therapeutic efficacy.

In our dataset, 4343 PL complexes with experimental *K*_*i*/*d*_ fall into SB and WB categories. This subset is used to classify SB and WB based on experimental vs MMPBSA and experimental vs docking binding affinities. For experimental binding affinities, the strong and weak binders were classified with a predefined cut-off value of −8.18 kcal/mol. The corresponding MMPBSA and docking cut-offs are −38.70 kcal/mol and −6.35 kcal/mol respectively. A brief discussion of the binding affinity cutoff values is given in detail in Supplementary Information.

**Fig. 3:**
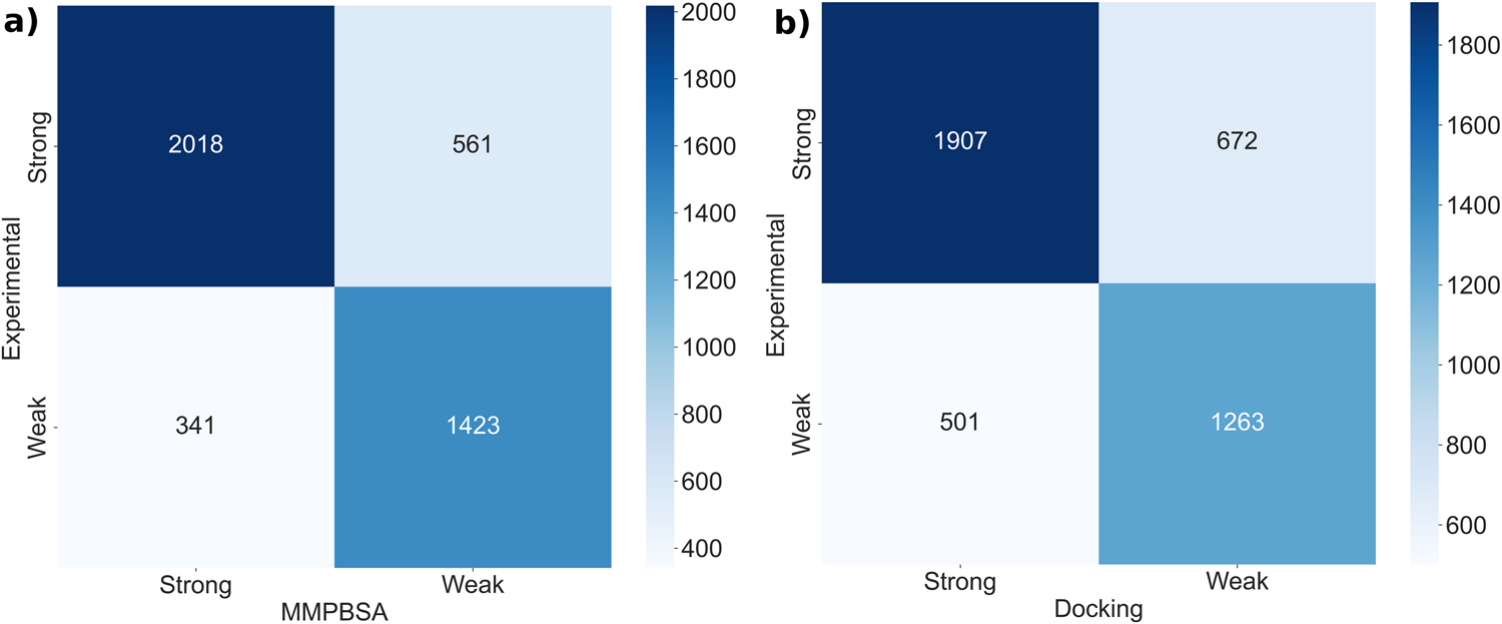
Confusion matrix to distinguish strong and weak binders (**a**) Experimental vs MMPBSA, (**b**) Experimental vs Docking.

The classification based on MMPBSA and Docking is shown in Fig. 3 and the qualitative performance was evaluated using the metrics given in Tables 1, 2. In Fig. 3, the diagonal elements of the confusion matrix represent the number of correct predictions, while the off-diagonal elements represent incorrect predictions. Based on the evaluation metrics, given in Tables 1, 2 and correlation coefficients (Supplementary Figure S5) it can be observed that MMPBSA classification is performing better compared to docking scores. Also, the confusion matrix revealed that the majority of SB (true positives) and WB (true negatives) were correctly identified with respect to MMPBSA, indicating the dataset is good enough to distinguish SB and WB. The definitions of the evaluation metrics are provided in SI.

**Table 1:** Performance metrics from confusion matrix to evaluate the classification models performance in distinguishing strong and weak binders based on MMPBSA calculations.

Exp. vs MMPBSA	Precision	Recall	f1-score	support
Strong Binders	0.86	0.78	0.82	2579
Weak Binders	0.72	0.81	0.76	1764
Accuracy			0.79	4343
Macro Average	0.79	0.79	0.79	4343
Weighted Average	0.80	0.79	0.79	4343

**Table 2:** Performance metrics from confusion matrix to evaluate the classification models performance in distinguishing strong and weak binders based on docking simulations.

Exp. vs Docking	Precision	Recall	f1-score	support
Strong Binders	0.79	0.74	0.76	2579
Weak Binders	0.65	0.72	0.68	1764
Accuracy			0.73	4343
Macro Average	0.72	0.73	0.72	4343
Weighted Average	0.74	0.73	0.73	4343

### Performance of Diverse Protein Sequences

The central goal of any machine learning (ML) model is to get the best model, and its performance depends on training data. More diverse the training data, one can expect a better model. We have collected a significantly large number of complex structures for this dataset preparation. Our dataset covers 1856 protein families which are of functional significance and a pie chart of the highly populated family is shown in supplementary Figure S6. Proteins with sequence similarity of ≤ 40% are grouped and the correlation coefficients are shown in Supplementary Figure S7. The results highlight the importance of the PLAS-20k dataset as it shows a good correlation for a diverse set of proteins.

### Performance Based on Ligand Structural Properties

In the field of drug discovery, prediction of bio-active molecules are based on several rules such as Lipinski^43^, MDDR-like rule^44^, Veber rule^45^, and Ghose filter^46^. The physicochemical properties like molecular weight and hydrogen bonding capacity are important to design drug-like molecules. For a comparison study, we chose a set of ligands with drug-like properties (Molecular weight ≤ 500, number of hydrogen bond donors ≤ 5, number of hydrogen bond acceptors ≤ 10) and evaluated the performance of those complexes based on docking and MMPBSA calculations.

**Fig. 4:**
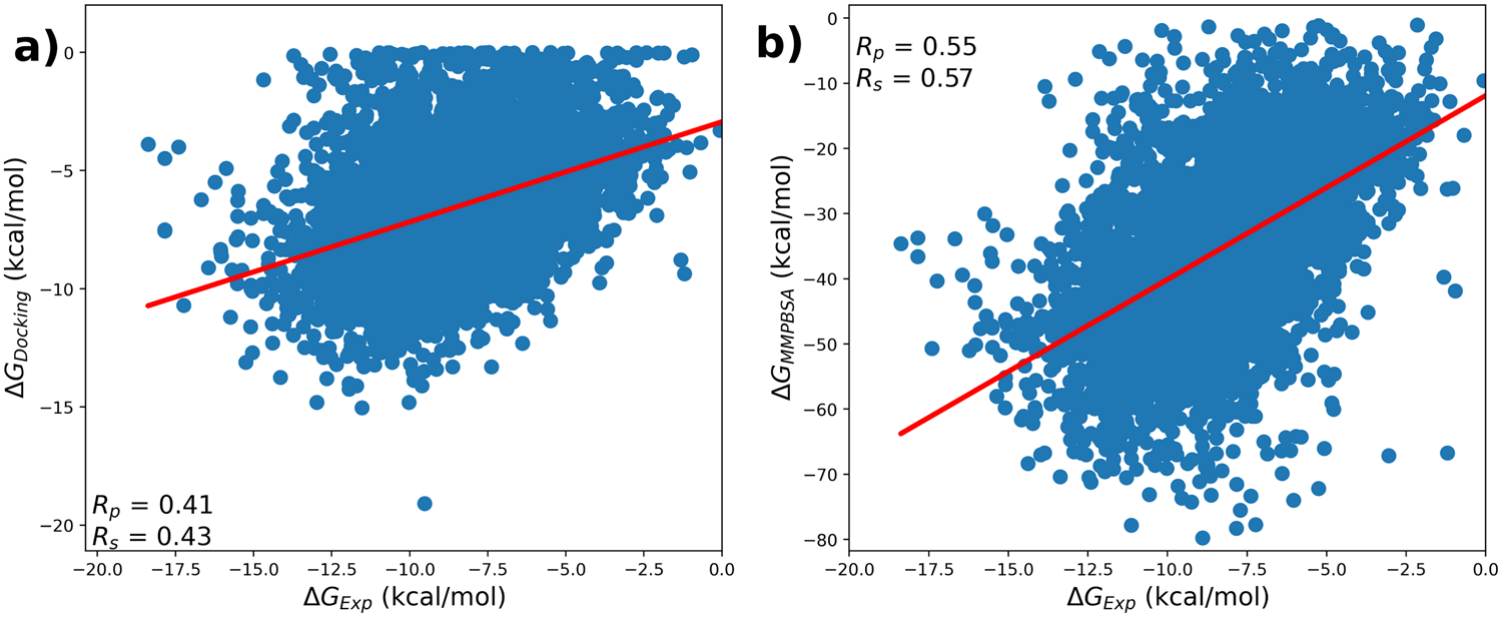
Correlation plots for a set of PDB ids from PLAS-20k (which follows Lipinski’s rule of five - Molecular weight, number of donors and number of acceptors of the ligand) for which experimental binding affinities are known - (**a**) Experimental vs Docking, (**b**) Experimental vs MMPBSA.

As seen in Fig. 4, MMPBSA calculations showed good correlation with (R_*p*_) of 0.55 and (R_*p*_) of 0.57 compared to docking with (R_*p*_),(R_*s*_) 0.41 and 0.43 respectively. Also, for each of the individual components of drug-like properties, MMPBSA showed a good correlation compared to docking and the results are shown in Supplementary Figure S8-S10. Further, as seen in Supplementary Figure S11 our dataset holds diverse ligands highlighting a few molecular descriptors, as they play an important role in drug discovery.

### Components of the Binding Free Energies

Binding free energy is the most important initial indicator of drug potency and remains a major challenge in predicting affinities. In this work, we have provided binding energies for 19,500 PL complexes along with energy components (Δ*E*_*ele*_, Δ*E*_*vdw*_, and Δ*G*_*Sol*_). This PLAS-20k dataset could be helpful in training ML models for predicting the binding affinities and energy components. The knowledge of these components can help in lead optimization. The distribution of the energy components is shown in Supplementary Figure S12. Moreover, the availability of dynamic binding poses from the PLAS-20k dataset can help in building ML models that can screen lead compounds in a more efficient manner compared to existing methods.

### Machine Learning Baseline

**Fig. 5:**
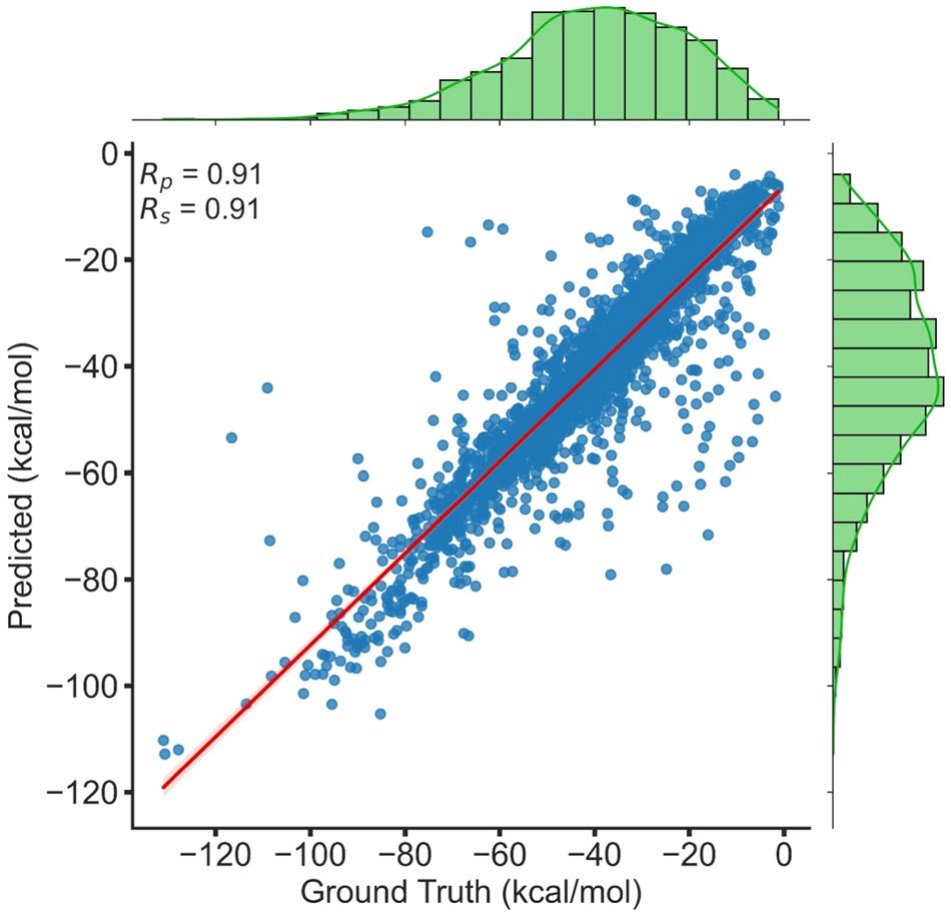
Pearson correlation coefficient of OnionNet trained on PLAS-20k dataset.

The prediction of binding affinity in the context of protein-ligand (PL) complexes plays a pivotal role in the field of drug design. Notably, machine learning (ML) methods have begun to significantly impact on this area. A noteworthy model in this domain is the innovative OnionNet. OnionNet operates by taking various features extracted from the three-dimensional molecular structure as input, coupled with known binding affinities. This information is then processed using a Convolutional Neural Network (CNN) to predict the binding affinity for unknown PL complexes. For the purpose of training and testing OnionNet, PLAS-20k data was utilized. To ensure the robustness of the model, a 10-fold cross-validation approach was employed. This technique involves dividing the dataset part (having corresponding experimental binding affinity counterparts) into ten equal components. Nine of the ten components have been used for training and the remaining one for testing. This approach is necessitated by the dataset′s size constraints. The model′s performance, as indicated by the average Root Mean Squared Error (RMSE) across all ten folds, stood at 8.15 kcal/mol. Furthermore, it demonstrated a strong correlation with an R_*p*_ value of 0.91, as depicted in Fig. 5. This further shows that the PLAS-20k dataset can be used effectively for training various ML and deep learning models.

### Supplementary information

Supplementary Information for: PLAS-20k: Extended Dataset of Protein-Ligand Affinities from MD simulations for Machine Learning Applications

## Data Availability

There is no in-house code used for ML model. We used OnionNet^47^ http://github.com/zhenglz/onionnet/ ML model to train on PLAS-20k dataset.
