# Author Correction: PLAS-20k: Extended Dataset of Protein-Ligand Affinities from MD Simulations for Machine Learning Applications

**DOI:** 10.1038/s41597-024-03585-6

**Published:** 2024-07-04

**Authors:** Divya B. Korlepara, C. S. Vasavi, Rakesh Srivastava, Pradeep Kumar Pal, Saalim H. Raza, Vishal Kumar, Shivam Pandit, Aathira G. Nair, Sanjana Pandey, Shubham Sharma, Shruti Jeurkar, Kavita Thakran, Reena Jaglan, Shivangi Verma, Indhu Ramachandran, Prathit Chatterjee, Divya Nayar, U. Deva Priyakumar

**Affiliations:** 1 IHub-Data, International Institute of Information Technology, Hyderabad, 500032 India; 2 Divison of Physics, School of Advanced Sciences, Vellore Institute of Technology, Chennai, 600127 India; 3 Department of Artificial Intelligence, School of Artificial Intelligence, Amrita Vishwa Vidyapeetham, Bengaluru, 560035 India; 4 Centre for Computational Natural Sciences and Bioinformatics, International Institute of Information Technology, Hyderabad, 500032 India; 5 Department of Materials Science and Engineering, Indian Institute of Technology Delhi, Hauz Khas, New Delhi 110016 India

**Keywords:** Data publication and archiving, Virtual screening

Correction to: *Scientific Data* 10.1038/s41597-023-02872-y, published online 09 February 2024

The original version of Figure 2 contained only the b) panel, incorrectly duplicated, instead of both a) and b).

Previous figure 2 
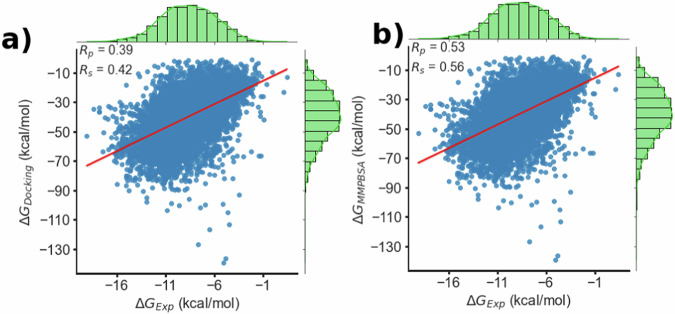


Correct figure 2 
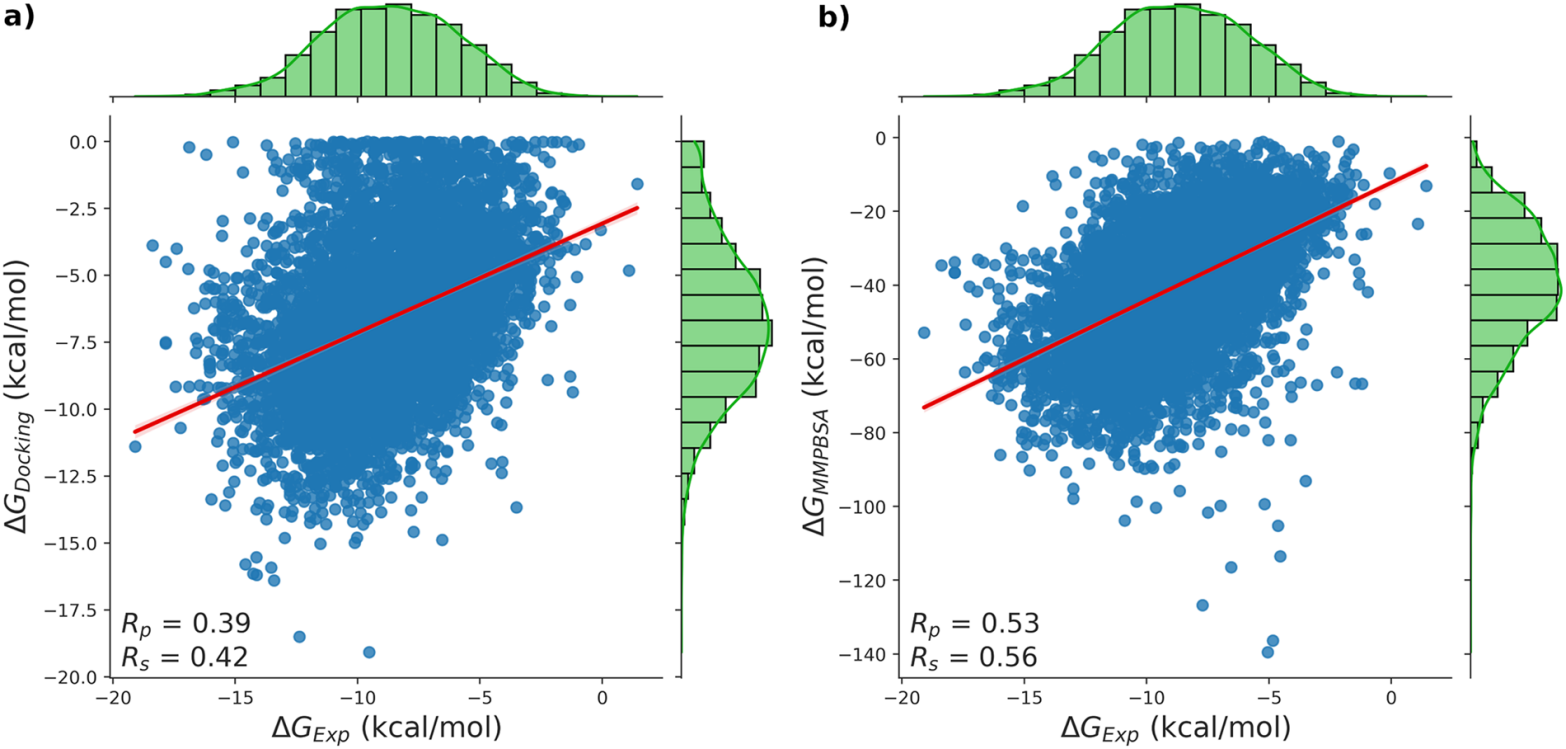


There was also an error in the Technical Validation section. In the original version we stated that a subset of 6842 complexes from PLAS-20k had experimental binding affinities whereas in fact this number was 6622. This error was made due to the original size of PLAS-20k being reduced from 20,000 to 19,500 complexes during the dataset creation but this reduction in size was not also accounted for in the validation sample. The correct (lower) sample size has been included in the manuscript (6842 replaced with 6622, where mentioned in the Technical Validation section and Figure 2 caption). Furthermore, the Pearson correlation coefficient (Rp) derived from this sample have been corrected to account for the correct sample size (originally stated as 0.50, now corrected to 0.53).

The dataset file at figshare has also been amended so the correct number of PLC data points are present, and to remove a duplicated file. The DOI for the previous version is 10.6084/m9.figshare.c.6742521.v1. The DOI for the updated files is 10.6084/m9.figshare.c.6742521.v2, which has been corrected in the data citation in the manuscript (reference 39).

Finally, two individuals were missing from the Acknowledgements section - Ms. Ananya Priya and Mr. Suyash Gupta. These individuals, along with the others currently listed, helped in data post-processing and website development.

All changes – the replacement of the figure, text changes, and change to the dataset DOI, have been applied to the pdf and HTML versions of the paper.

