# Supplementary material for: PLAS-20k: Extended Dataset of Protein-Ligand Affinities from MD Simulations for Machine Learning Applications
Source: Sci Data. 2024 Feb 9;11:180. doi: 10.1038/s41597-023-02872-y (PMC10858175; doi:10.1038/s41597-023-02872-y)
Supplement: Supplementary file 1 — Supplementary Information for: PLAS-20k: Extended Dataset of Protein-Ligand Affinities from MD simulations for Machine Learning Applications [file 41597_2023_2872_MOESM1_ESM.pdf]

Supplementary Information for: PLAS-20k: Extended Dataset of Protein-Ligand Affinities from MD simulations for Machine Learning Applications

Divya B. Korlepara[1,3], Vasavi C. S.[1,4], Rakesh Srivastava[2], Pradeep Kumar Pal[2], Saalim H. Raza [1], Vishal Kumar[2], Shivam Pandit[1], Aathira G Nair[1], Sanjana Pandey[1], Shubham Sharma[1], Shruti Jeurkar[2], Kavita Thakran[1], Reena Jaglan[1], Shivangi Verma[1], Indhu Ramachandran[1], Prathit Chatterjee[1],Divya Nayar[5,*], and U. Deva Priyakumar[1,2,*]

[1]*IHub-Data, International Institute of Information Technology, Hyderabad, 500032, India.*

[2]*Centre for Computational Natural Sciences and Bioinformatics, International Institute of Information Technology, Hyderabad, 500032, India.*

[3]*Divison of Physics, School of Advanced Sciences, Vellore Institute of Technology, Chennai, 600127, India.*

[4] *Department of Artificial Intelligence, School of Artificial Intelligence, Amrita Vishwa Vidyapeetham, Bengaluru, 560035, India.*

[5]*Department of Materials Science and Engineering, Indian Institute of Technology Delhi, Hauz Khas, New Delhi, 110016, India.*

E-mail: divyanayar@mse.iitd.ac.in,deva@iiit.ac.in

Contents

Figure S1: Screenshot of the PDB Viewer **7**

Figure S2: Nonlinear t-SNE distributions of PLAS-5k, and PLAS-20k for the molecular properties obtained from ligand-SMILES strings. The green circles represent the t-SNE points corresponding to PLAS-20k data, whereas the magenta squares represent corresponding PLAS-5k subset. **8**

Figure S3: The distribution of RMSD of the protein and ligand from molecular dynamics simulations. **8**

Figure S4: The distribution of binding affinities computed for 14500 protein-ligand complexes using MMPBSA method. **9**

Figure S5: The correlation plot for PL complexes (4343) with experimental binding affinities under weak and strong binders. a) Experimental vs MMPBSA and b) Experimental vs Docking **9**

Figure S6: The population of each family of proteins in PLAS-20k dataset **10**

Figure S7: Correlation plots between the experimental and calculated binding affinities for a subset with 1185 (protein sequence with $\leq$ 40%) pdbids. The calculated binding affinities are calculated (a) using Docking, and (b) using MMPBSA. **10**

Figure S8: Correlation plots for a set of pdbids from PLAS-20k (Molecular weight of ligand as per Lipinski rule of five) for which experimental binding affinities are known - (a) Experimental vs Docking, (b) Experimental vs MMPBSA. **11**

Figure S9: Correlation plots for a set of pdbids from PLAS-20k (Number of

hydrogen bond acceptors of ligand as per Lipinski rule of five) for which experimental binding affinities are known - (a) Experimental vs Docking, (b) Experimental vs MMPBSA. 11

Figure S10: Correlation plots for a set of pdbids from PLAS-20k (Number of hydrogen bond donors for ligand as per Lipinski rule of five) for which experimental binding affinities are known - (a) Experimental vs Docking, (b) Experimental vs MMPBSA. 12

Figure S11: The distribution of the descriptors of the ligand of PLAS-20k PL complexes, a) No. of amide Bonds, b) No. of aromatic carbocycles, c) No. of aromatic rings, d) No. of rings, e) No. of rotatable bonds and f) molecular weight. 13

Figure S12: The distribution of calculated energy components of binding affinity from MMPBSA method (a) Electrostatic, (b) van der Waals, (c) Non-polar Solvation free energy and (d) Polar Solvation free energy for 14500 protein-ligand complexes. 14

Binding Affinity - Cutoff

Equations (1),(2) provide the experimental binding affinity of -9.54 kcal/mol for an SB with a binding constant k_i of 10 nM and -6.82 kcal/mol for a WB for a binding constant k_i of 100 μM respectively.

$$B.E_{exp}^{strong} = -9.54\ kcal/mol\ (k_i = 10\ nM) \tag{1}$$

$$B.E_{exp}^{weak} = -6.82\ kcal/mol\ (k_i = 100\ \mu M) \tag{2}$$

Equation (3) introduces the concept of a cutoff value that can be used to classify binding affinities as either strong or weak. The cutoff value is determined by taking the average of the binding affinities for SB and WB and the calculated cutoff value $Cutoff_{exp}$ is -8.18 kcal/mol.

$$Cutoff_{exp} = \frac{B.E_{exp}^{weaker} + B.E_{exp}^{strong}}{2} = -8.18\ kcal/mol \tag{3}$$

Equation (4) represents a linear regression line where Y_{exp}^{MMPBSA} is determined as a function of X. The regression line is derived from pairwise correlation between experimental and MMPBSA binding affinities. The cut-off values for SB and WB are estimated using (Y_{exp}^{MMPBSA}).

$$Y_{exp}^{MMPBSA} = -12.94 + 3.15 * (X),\ \text{where Y is the regression line} \tag{4}$$

In Equations (5),(6) for experimental binding affinities of -9.54 and -6.82 kcal/mol, the estimated MMPBSA values are -42.99 kcal/mol (SB) and -34.33 kcal/mol (WB) respectively. The corresponding correlation plot is shown in Fig. S5b

$$B.E_{MMPBSA}^{strong} = -12.94 + 3.15 * (-9.54) = -42.99\ kcal/mol \tag{5}$$

$$B.E_{MMPBSA}^{weak} = -12.94 + 3.15 * (-6.82) = -34.423 \; kcal/mol \tag{6}$$

Equation (7) represents the calculation of the MMPBSA cut off value to distinguish strong and weak binders. PL complexes with MMPBSA afinities above the cuttoff value -38.70 *kcal/mol* are classified as SB and binders with MMPBSA affinities below the cutoff value are classified as WB.

$$Cutoff_{MMPBSA} = \frac{B.E_{MMPBSA}^{weaker} + B.E_{MMPBSA}^{strong}}{2} = -38.70 \; kcal/mol \tag{7}$$

A similar procedure as MMPBSA cut-off was applied in estimating the docking cut-off and the regression equation based on docking affinities is given in Equation (8)

$$Y_{exp}^{Docking} = -3.08 + 0.40 * (X) \text{where Y is the regression line} \tag{8}$$

In Equations (9),(10) for experimental binding affinities of -9.54 and -6.82 kcal/mol, the estimated Docking values are -6.90 kcal/mol (SB) and -5.80 kcal/mol (WB) respectively. The corresponding correlation plot is shown in Fig. S5a

$$B.E_{Docking}^{strong} = -3.08 + 0.40 * (-9.54) = -6.896 \; kcal/mol \tag{9}$$

$$B.E_{Docking}^{weak} = -3.08 + 0.40 * (-6.82) = -5.80 \; kcal/mol \tag{10}$$

Equation (11) represents the calculation of the docking cut off value to distinguish strong and weak binders. PL complexes with Docking afinities above the cuttoff value -6.352 *kcal/mol* are classified as SB and binders with Docking affinities below the cutoff value are classified as WB.

$$Cutoff_{Docking} = \frac{B.E_{Docking}^{weaker} + B.E_{Docking}^{strong}}{2} = -6.352 \; kcal/mol \tag{11}$$

Evaluation Metrics

Precision:

Precision is a measure of how accurately a classifier predicts a class. It is computed as the ratio of correctly predicted positives to the total predicted positives.

$$Precision = \frac{True\ positives}{True\ positives + False\ positives}$$

Accuracy:

Accuracy is a measure of how accurately a classifier can predict both positive and negative classes. It is computed as the ratio of correctly predicted samples to the total number of samples.

$$Accuracy = \frac{True\ positives + True\ negatives}{True\ positives + True\ negatives + False\ positives + False\ negatives}$$

F1-Score:

F1-Score is a measure of a classifier's accuracy, taking into account both precision and recall. It is computed as the harmonic mean of precision and recall.

$$F1 - Score = \frac{2 * (Precision * Recall)}{(Precision + Recall)}$$

Support:

Support is the number of samples belonging to a particular class.

$$Support = True\ positives\ + True\ negatives + False\ positives\ + False\ negatives$$

Macro Average:

Macro average is the unweighted mean of the precision, recall, and other scores for each class.

$$Macro\ Average = \sum_{i=1}^{N} \frac{(Score\ for\ class\ i)}{N}$$

Weighted Average:

Weighted average is the weighted mean of the precision, recall, and other scores for each class, with the weights being the number of samples belonging to each class.

$$Weighted\ Average = \frac{\sum_{i=1}^{N}(Score\ for\ class\ i * Weight\ for\ class\ i)}{\sum_{i=1}^{N} Weight\ for\ class\ i}$$

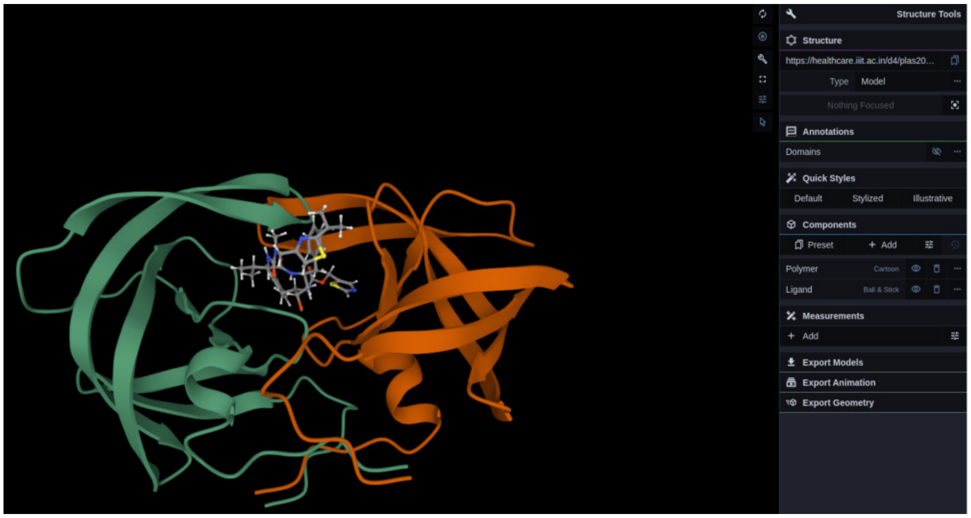

Figure S1: Screenshot of the PDB Viewer

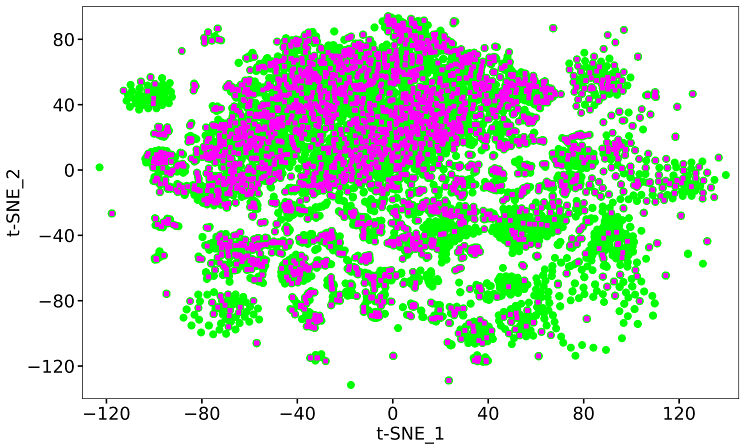

Figure S2: Nonlinear t-SNE distributions of PLAS-5k, and PLAS-20k for the molecular properties obtained from ligand-SMILES strings. The green circles represent the t-SNE points corresponding to PLAS-20k data, whereas the magenta squares represent corresponding PLAS-5k subset.

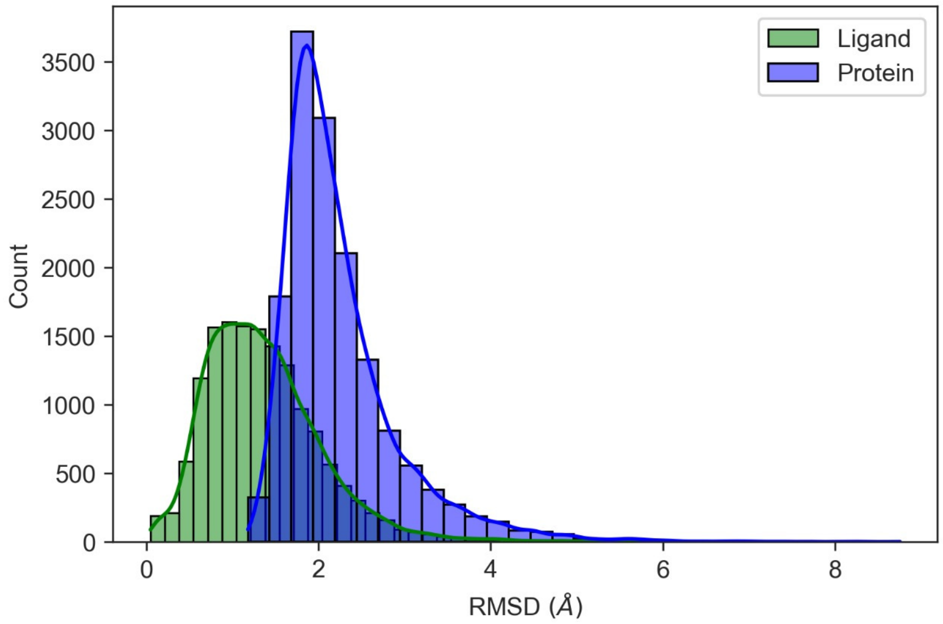

Figure S3: The distribution of RMSD of the protein and ligand from molecular dynamics simulations.

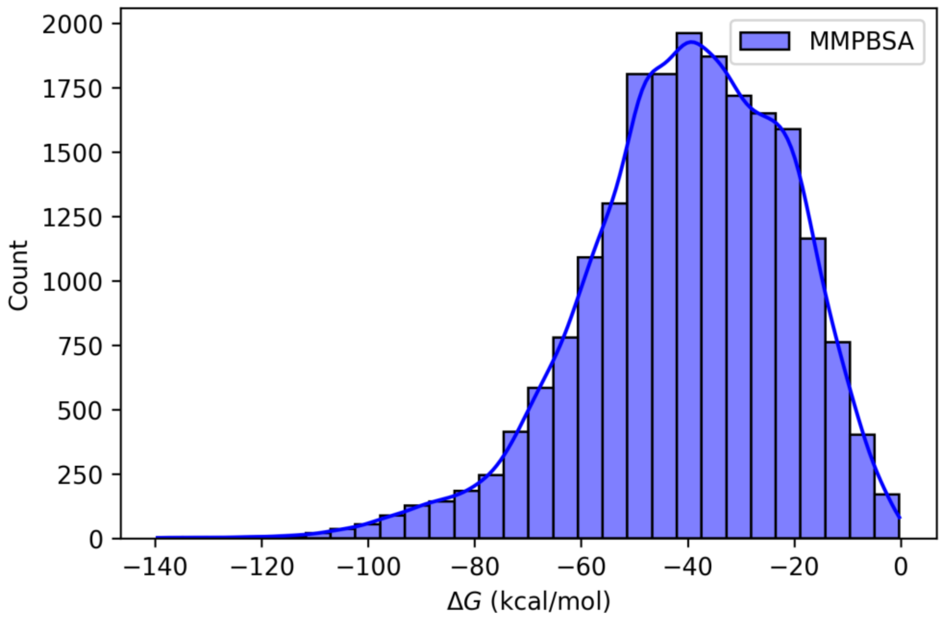

Figure S4: The distribution of binding affinities computed for 14500 protein-ligand complexes using MMPBSA method.

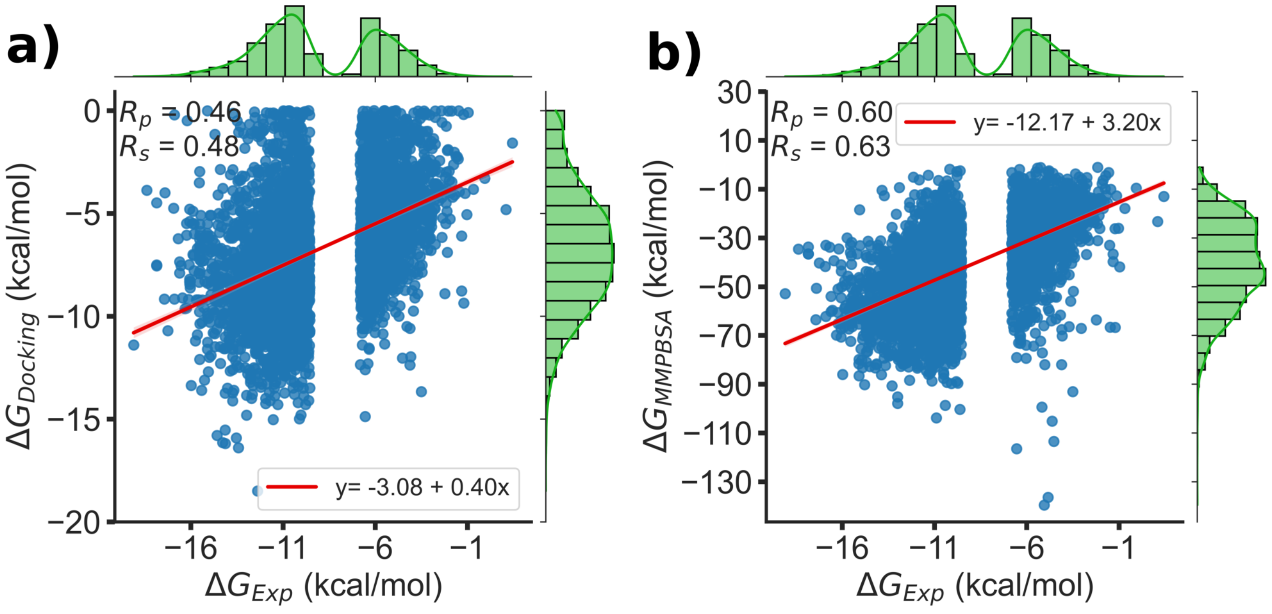

Figure S5: The correlation plot for PL complexes (4343) with experimental binding affinities under weak and strong binders. a) Experimental vs MMPBSA and b) Experimental vs Docking

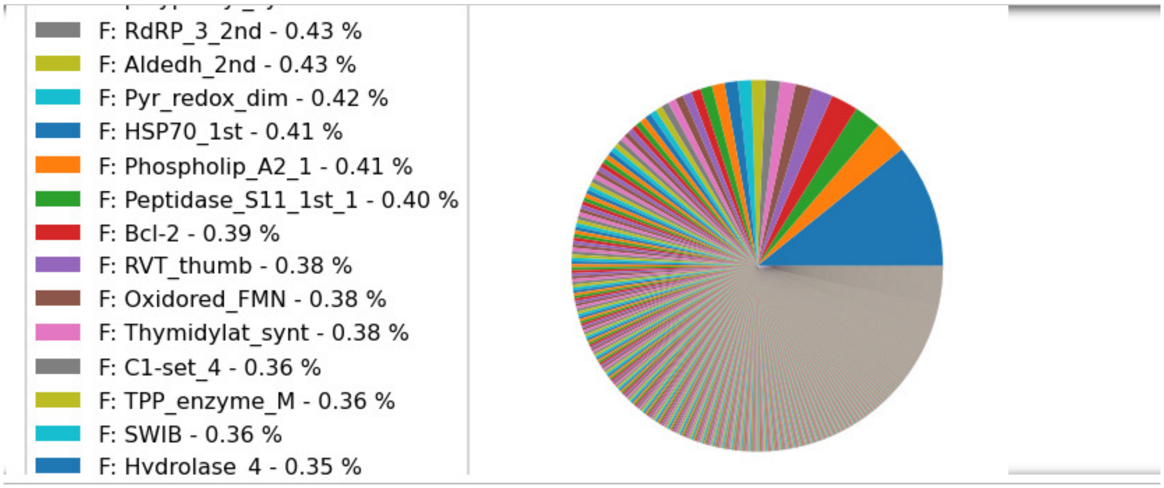

Figure S6: The population of each family of proteins in PLAS-20k dataset

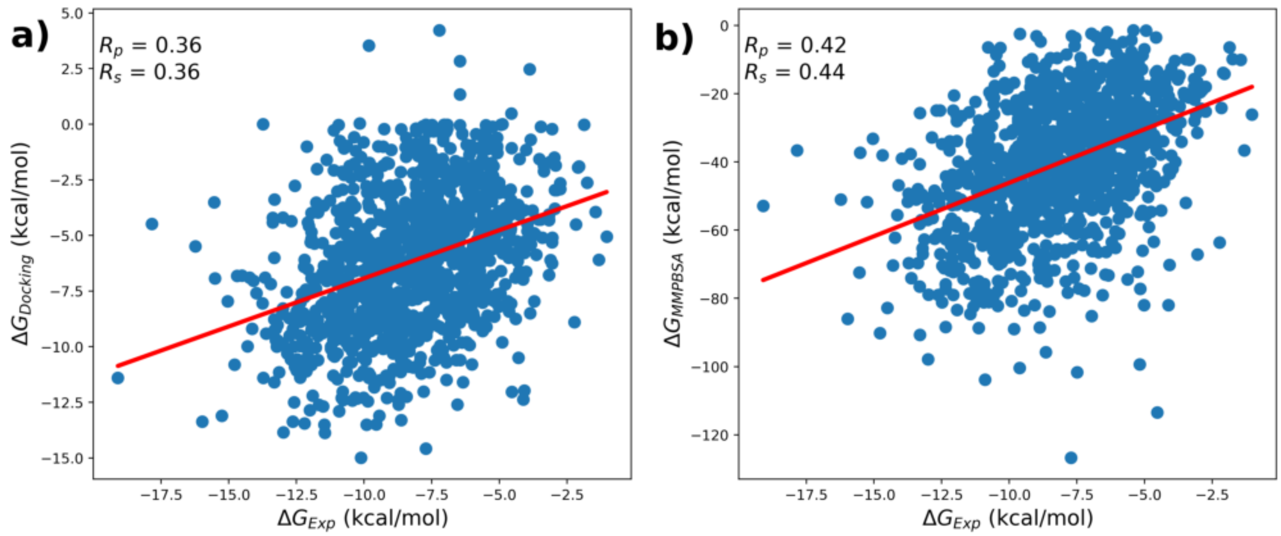

Figure S7: Correlation plots between the experimental and calculated binding affinities for a subset with 1185 (protein sequence with $\leq 40\%$) pdbids. The calculated binding affinities are calculated (a) using Docking, and (b) using MMPBSA.

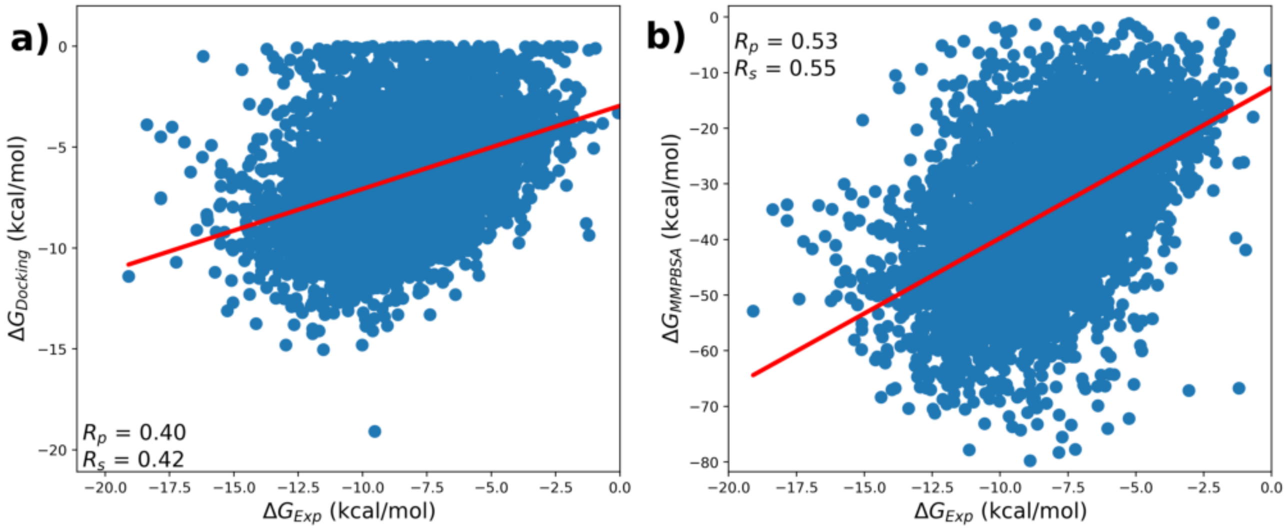

Figure S8: Correlation plots for a set of pdbids from PLAS-20k (Molecular weight of ligand as per Lipinski rule of five) for which experimental binding affinities are known - (a) Experimental vs Docking, (b) Experimental vs MMPBSA.

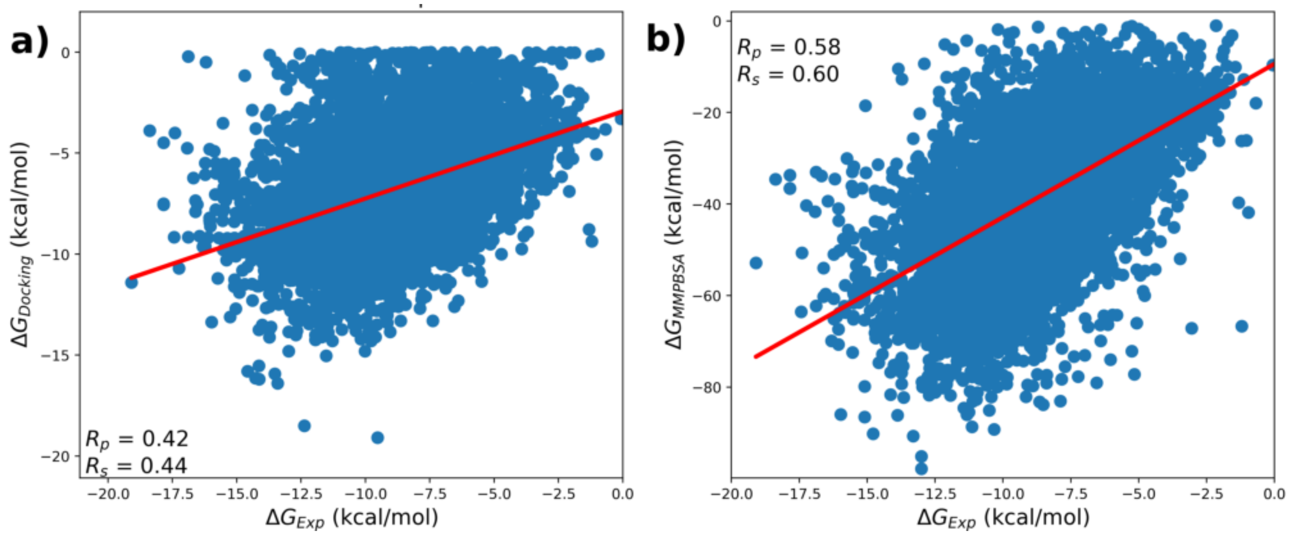

Figure S9: Correlation plots for a set of pdbids from PLAS-20k (Number of hydrogen bond acceptors of ligand as per Lipinski rule of five) for which experimental binding affinities are known - (a) Experimental vs Docking, (b) Experimental vs MMPBSA.

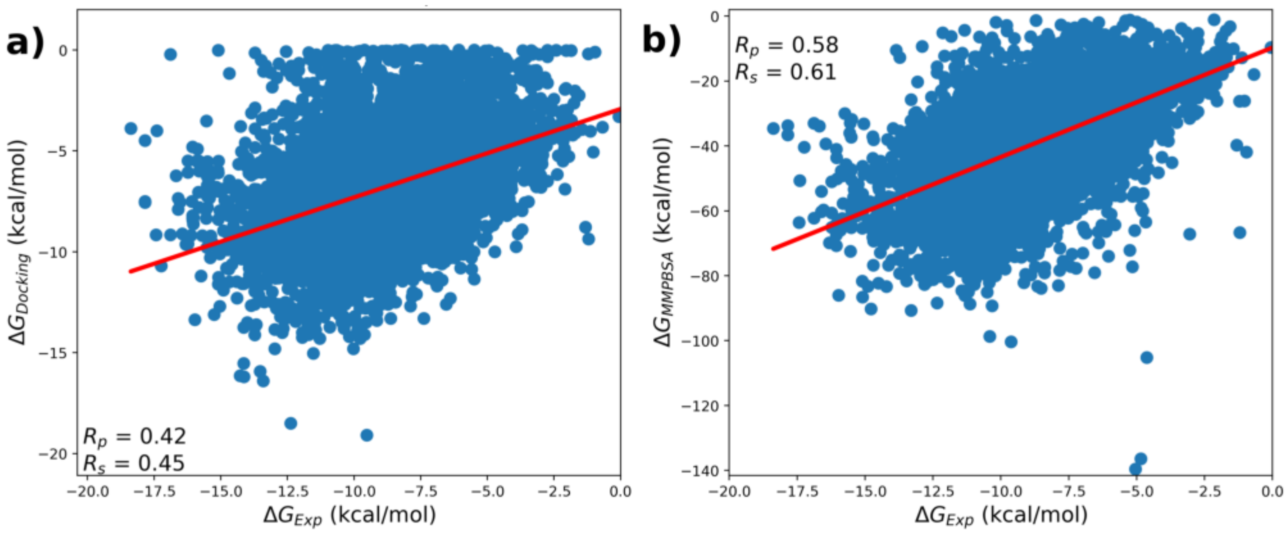

Figure S10: Correlation plots for a set of pdbids from PLAS-20k (Number of hydrogen bond donors for ligand as per Lipinski rule of five) for which experimental binding affinities are known - (a) Experimental vs Docking, (b) Experimental vs MMPBSA.

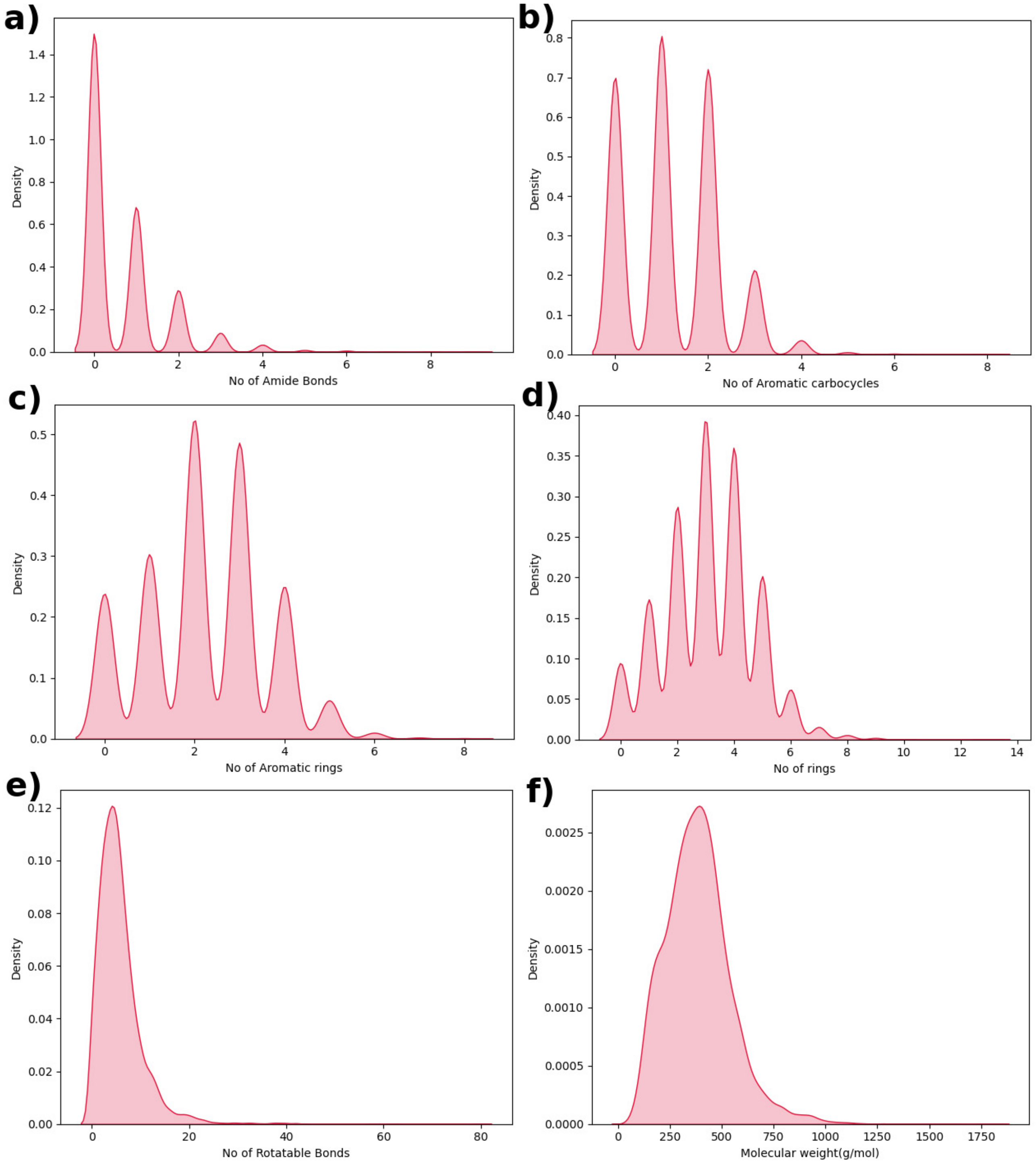

Figure S11: The distribution of the descriptors of the ligand of PLAS-20k PL complexes, a) No. of amide Bonds, b) No. of aromatic carbocycles, c) No. of aromatic rings, d) No. of rings, e) No. of rotatable bonds and f) molecular weight.

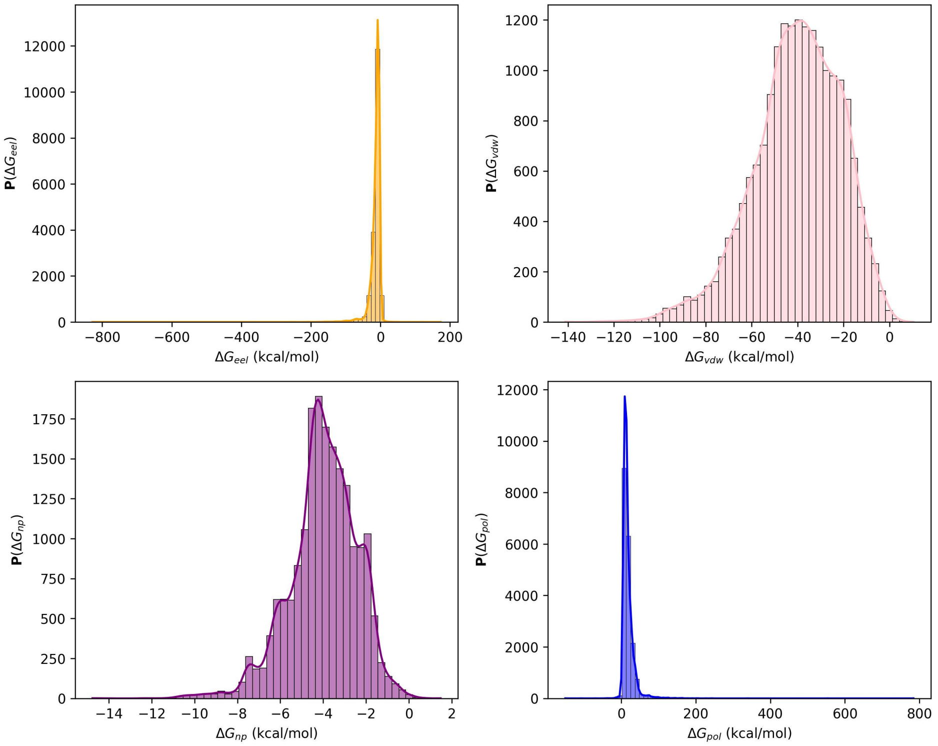

Figure S12: The distribution of calculated energy components of binding affinity from MMPBSA method (a) Electrostatic, (b) van der Waals, (c) Non-polar Solvation and (d) Polar Solvation for 14500 protein-ligand complexes.
